# Wound leakage in the early postoperative phase correlates with surgical site infections following total hip and knee arthroplastry, a case control study

**DOI:** 10.1186/2047-2994-4-S1-P69

**Published:** 2015-06-16

**Authors:** S Camps, K Kremers-van de Hei, S Köeter, A Tostmann, M Nabuurs-Franssen, A Voss

**Affiliations:** 1 Infection Prevention, Canisius-Wilhelmina Hospital, Nijmegen, Netherlands; 2 Orthopaedics, Canisius-Wilhelmina Hospital, Nijmegen, Netherlands; 3 Infection Prevention, Radboud University Medical Centre, Nijmegen, Netherlands

## Introduction

A Surgical Site Infection (SSI) following total hip or knee arthroplasty (THA/TKA) is considered to be a devastating complication, leading to prolonged hospitalization, repeated surgical intervention and often the removal of the prosthesis.

## Objectives

We performed a study to investigate which factors were associated with the development of a SSI following THA/TKA, in order to identify leads for reducing the risk of SSI.

## Methods

We identified 25 cases who developed SSI following THA/TKA. For each case we included two controls matched on type of surgery (THA/TKA) and date/time of surgery (i.e. surgery before- and after the case). Analysed variables included age, gender, body mass index, known co-morbidities (such as diabetes and smoking), preoperative length of stay, ASA-classification, antibiotic prophylaxis, therapeutic anticoagulation, *S. aureus* decolonization, use of drains, wound closing, normothermia, haematoma, wound leakage, dressing changes and length of stay.

## Results

Cases had significantly more often co-morbidities, hematoma, wound leakage and wound dressing change compared to controls. Noticeable, in cases the wound dressing changes occurred more often on the day of surgery, as well as at day one and day two postoperative. Cases also had a prolonged postoperative stay in the hospital. For the other variables, there was no statistically significant difference.

## Conclusion

The study was undertaken after an unexplained increase in SSI, following a period of multiple changes, including the implementation of fast-track surgery. It is likely that formation of hematoma as well as increased wound leakage, lead to the increased risk of SSI. In literature, persistent wound leakage (>72 hours) is linked to development of SSI. Surprisingly, we found more wound leakage in the early postoperative phase correlated with SSI. The use of tranexamic acid, suction drains and a critical evaluation of guidelines for preventing thromboembolic events all offer reducing the risk on wound leakage and the development of SSI.

## Disclosure of interest

None declared.

